# Lower left atrial function in young individuals with type 1 diabetes mellitus compared to healthy controls: an echocardiographic study

**DOI:** 10.1038/s41598-024-54597-6

**Published:** 2024-02-17

**Authors:** Cecilia Fridolfsson, Johanna Thegerström, Karin Åkesson, Jan Engvall, Peter Blomstrand

**Affiliations:** 1 Department of Clinical Physiology in Kalmar, Region Kalmar County, Kalmar, Sweden; 2 Department of Health, Medicine and Caring Sciences, Linköping University, Linköping, Sweden; 3 Department of Paediatrics in Kalmar, Region Kalmar County, Kalmar, Sweden; 4 Faculty of Health and Life Sciences (FHL), Linnaeus University, Kalmar, Sweden; 5 Department of Pediatrics, Ryhov County Hospital, Jönköping, Sweden; 6 Department of Clinical Physiology in Linköping, Linköping University Hospital, Linköping, Sweden; 7 Department of Natural Sciences and Biomedicine, School of Health and Welfare, Jönköping University, Jönköping, Sweden

**Keywords:** Cardiology, Endocrinology

## Abstract

In adulthood, individuals with type 1 diabetes mellitus may develop a condition of heart failure with preserved ejection fraction. However, subclinical changes to the heart in diabetes are likely to occur prior to the clinical presentation. This cross-sectional study aimed to compare left atrial function by echocardiography between 43 individuals with type 1 diabetes and 43 healthy controls, aged 10–30 years. All participants underwent echocardiography and 2D speckle tracking measurements for left atrial phase function parameters. Physical capacity was assessed by exercise test on a bicycle. Results showed that participants with type 1 diabetes had significantly lower left atrial function parameters than healthy controls (p < 0.05). There was a significant negative correlation between HbA1c means and reservoir and conduit strain (p < 0.05) and individuals with BMI < 30 showed a lower left atrial stiffness (p < 0.05). Individuals with type 1 diabetes and a higher physical capacity did not differ from their healthy peers. Results indicate that lower HbA1c levels, BMI < 30 and a higher physical capacity are favourable in terms of left atrial function in children and young adults with type 1 diabetes mellitus. Left atrial strain by echocardiography might become a new important tool in assessing heart function in T1DM.

## Introduction

Diabetes duration and glycaemic control are important risk factors for cardiovascular disease in type 1 diabetes mellitus (T1DM)^1^, as poor metabolic control contributes to the development of chronic micro- and macrovascular complications^2–7^. Other modulating factors that might contribute to disease progression are sedentary behaviour^8^, overweight/obesity and elevated blood pressure^9–12^. Contrarily, physical activity will contribute to better metabolic control and delay the development of cardiovascular disease^1,13,14^.

One of the cardiovascular complications in T1DM is heart failure with preserved ejection fraction (HFpEF)^15^, sometimes labelled early-stage diabetic cardiomyopathy (DCM), in which patients with diabetes develop subclinical heart failure without the presence of arterial hypertension or myocardial ischemia due to coronary artery disease^15–17^. The pathogenesis of this condition involves microangiopathy of the small vessels of the heart, progressively leading to myocardial fibrosis, dysfunctional remodelling of heart chambers and, over time, left ventricular dysfunction^18–21^. Impaired diastolic cardiac function is an early sign of DCM and usually manifests before left ventricular (LV) systolic dysfunction^3,5,17,22^, which is why it is pertinent to investigate diastolic dysfunction early in young individuals with T1DM^23^.

Recent studies indicate that the left atrium (LA) plays a key role in diastolic function, as the LA modulates the filling of the left ventricle (LV)^24,25^ and is considered to actively contribute to symptoms of HFpEF^5,26^. Hemodynamics in general and the diastolic function of the heart in particular, are modulated by the LA at the filling of the LV, through several steps called reservoir, conduit and contractile (boost pump) phasic functions^27^.

Echocardiography is the most frequently used method to assess cardiac function and morphology. Left atrial strain measured by speckle tracking echocardiography is a method to assess atrial function at a detailed level^24,25^. The analysis is not performed routinely in the clinical setting, but is proposed to become part of the assessment of diastolic function including left atrial function and stiffness in the future^27–30^.

Early signs of cardiovascular changes are difficult to measure. Currently, there is no common screening program for cardiovascular complications in diabetes in the young, equivalent to the screening for nephro- and retinopathy. Improved methods for monitoring of the cardiovascular system may be beneficial for individuals with diabetes in the prevention of heart failure^7,31^.

The primary aim of the present study was to compare left atrial function by echocardiography in adolescents and young adults with T1DM to that of healthy controls. The hypothesis was that left atrial phase function assessed by echocardiography would be reduced in adolescents and young adults with T1DM in comparison to healthy controls.

The secondary aim was to investigate modulating factors of left atrial function, such as metabolic control, physical work capacity, body mass index (BMI) and blood pressure, in adolescents and young adults with T1DM.

## Methods

### Study population

This is a sub-study of the Heart Rate Variability in type 1 diabetes study (HRV-D study), which investigates cardiac autonomic function from 24 h-ambulatory electrocardiograms (Holter ECG). Participants between 10 and 30 years of age were consecutively enrolled from the diabetes clinics for children and adults in Kalmar, Jönköping and Västervik, Sweden. The control group, matched for age and gender, consisted of individuals who were recruited either as a friend of the participants with T1DM (but not sibling), or from primary and secondary schools in Kalmar, Sweden, by school nurses. In total, 43% of eligible participants from the original study chose to enrol in this in-depth, echocardiographic study of LA-function.

Exclusion criteria comprised the presence of cardiovascular disease, neurological disease, and systemic disease except from T1DM, ongoing depression, sleep disorder or renal failure. Some of the study participants with T1DM had a mild form of retinopathy, but no other known significant diabetes complications. Three adult individuals with T1DM were treated with statins (7% of study population). Basic data in terms of age, gender, height, weight, blood pressure and resting heart rate were collected at the echocardiographic examination. BMI was calculated from weight and hight; for children, BMI was converted to isoBMI according to WHO^32^. Duration of diabetes and HbA1c data from onset of diabetes to study examination were retrieved from the Swedish National Diabetes Register (NDR). From the onset of diabetes, HbA1c was measured and reported to NDR every three months until the age of 18, and after 18 years of age, it was measured and reported once or twice a year. Participants did not differ in metabolic control (HbA1c levels) from those who declined to participate. The HbA1c samples were analysed at the chemistry lab at Jönköping and Kalmar County hospitals, which are accredited according to ISO-IEC 15189.

### Ethics approval and consent to participate

Written informed consent was obtained from participants 18 years of age and older, and if younger than 18 years, also from the participants’ guardians. The study was approved by the regional ethical review board in Linköping, Sweden, (2018/169–32). and was conducted in accordance with the principles stated in the Declaration of Helsinki.

### Technical facilities

Laboratory facilities at the Department of Clinical Physiology at Kalmar County Hospital and at Jönköping County Hospital and at the Department of Internal Medicine at Västervik Hospital were used. All transthoracic echocardiograms were performed using the same brand of ultrasound equipment (Vivid E9 and E95 GE Healthcare) in all three locations. Images were analysed offline using the software Echo PAC 204, GE Healthcare, at the Department of Clinical Physiology, Kalmar County Hospital. The exercise tests were carried out in Kalmar and Västervik using ECG equipment GE CASE V6.73 and Ergometer cycle Rodby RE 990. In Jönköping ECG equipment GE CASE V6.61 (2018) and V7.0 (2022) and Ergometer cycle Rodby RE990 were used. Ergometer bicycles were regularly calibrated by the certified vendor.

### Echocardiographic protocol

The transthoracic echocardiograms were performed by an experienced examiner (echocardiography technician with professional experience > 25 years). Parasternal long- and short-axis views and apical standard four-chamber (4-Ch), two-chamber (2-Ch), and three-chamber (3-Ch) views were obtained^33^.

The examination included conventional and detailed echocardiography of cardiac function with 2D imaging, Colour Doppler, Pulsed Wave Doppler, and Tissue Doppler imaging (TDI). Three consecutive cardiac cycles were acquired in cine format in all views, during a breath hold at end‐expiration. The 2D images were acquired at as high frame rate as possible (> 40 frames per second (fps)) and care was taken to include the entire left atrium in the apical images to optimize speckle tracking (STE) for further analysis.

### Echocardiographic assessment

LA maximum volume (LAmax) was measured at end systole, using a modified Simpson's biplane formula^33^. An average value for each volume was obtained from three cardiac cycles and indexed to body surface area (BSA). LA stiffness was calculated as E/e' (average of medial, lateral)/LA reservoir strain %. LV end-diastolic volume (EDV), LV end-systolic volume (ESV) and left ventricle ejection fraction (LVEF) were calculated by Simpson's biplane method. The measurements for chamber quantification were assessed as recommended by the American Society of Echocardiography and the European Association of Cardiovascular Imaging^33^.

Mitral inflow velocity early peak (E-wave), E-wave deceleration time (DT) and atrial contraction (A-wave) were obtained with pulsed wave (PW) Doppler at end-expiration, including E/A ratio to assess diastolic filling of the left ventricle. Pulsed wave tissue Doppler septal and lateral velocities of the mitral annulus were obtained at end-expiration, to define early (e') and late (a') diastolic peak and systolic (s') peak velocities. Average septal and lateral e' were used for calculation of E/e'. As an additional measurement to evaluate diastolic filling of the left atrium, the pulmonary venous flow systole (S)/diastole (D) ratio was obtained with PW Doppler at end-expiration. The diastolic measurements were assessed as recommended by the American Society of Echocardiography and the European Association of Cardiovascular Imaging^33^.

### Left atrium strain analysis with two-dimensional (2D) speckle tracking (STE)

**Figure 1:**
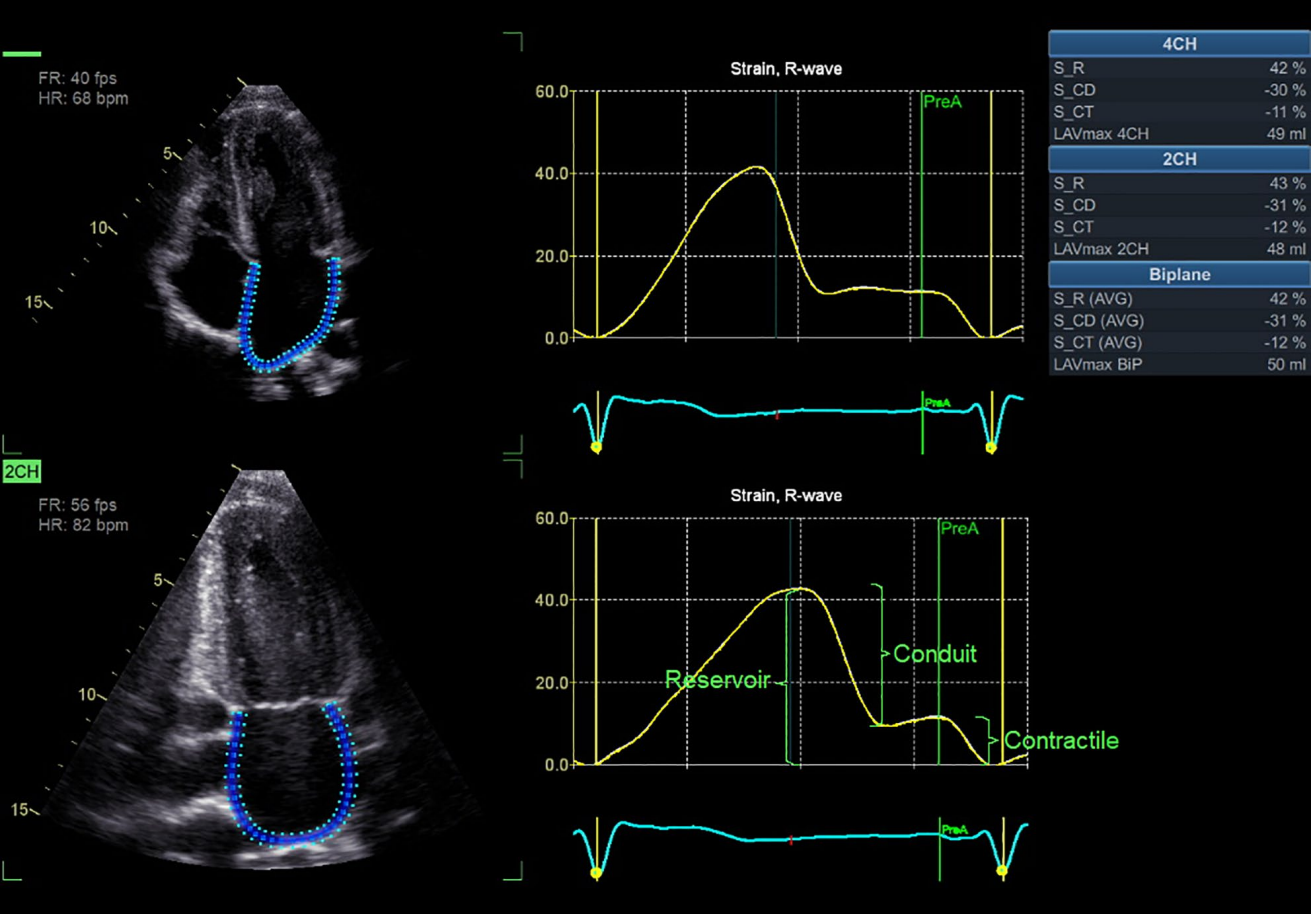
Strain analysis in 4- and 2-ch views, with the different phasic functions of LA strain (S): Peak atrial reservoir (S_R), conduit (S_CD) and contractile strain (S_CT) are presented in the table (upper right corner) for 4- and 2-ch views separately as well as a biplane average (Photo. Fridolfsson C).

2D speckle tracking: strain analysis of the LA was obtained from 2D images. Three cardiac cycles were recorded in both apical 4- and 2-ch views with particular care to avoid LA- foreshortening^5^. LA strain was analysed using GE Healthcare EchoPAC 204 software Automated Functional Imaging (AFI) LA, which is a 2D STE technology. LA strain was calculated from the onset of the R wave, and as the mean of three valid measurements. AFI LA is a semi-automatic analysis performed in apical 4- and 2-ch views. The region of interest was traced automatically by the software along the endocardial border in end-systole. When the alignment proved unsatisfactory, the tracing was edited manually until correct alignment of the left atrium was achieved. The strain curve was displayed after data processing. Three phases of LA strain (S) were identified: peak atrial reservoir (S_R), conduit (S_CD) and contractile strain (S_CT). The results were calculated for 4- and 2-ch views separately and as an average. The definition of LA reservoir, conduit and contractile function is demonstrated in Fig. 1.

### Exercise testing

The exercise tests were performed on a cycle ergometer with adjustable electronic resistance with a continuous ramp protocol in order to measure maximum workload in watts (W). A standardized research protocol with an initial load of 30 W was used. The load was increased by 10 W/min in women and children/adolescents up to 16 years of age, and by 20 W/min in men (> 16 years). All participants performed the test to maximum effort (Borg scale 17 or higher, and heart rate at maximum exercise > 85% of predicted) and the results were presented in Watts and as the percentage of predicted maximum workload for age, sex and BSA^34^.

### Categories

Participants were dichotomized according to sex and age with a cut-off at 18 years, to obtain a paediatric group and an adult group. The participants with T1DM were dichotomized according to duration of diabetes in similar sized groups with a cut-off at 10 years, as well as according to HbA1c level with a cut off at 48 mmol/mol, since this is the official (although debated) treatment goal of the Swedish Society for Paediatric Endocrinology and Diabetes, based on International Society for Pediatric and Adolescent Diabetes (ISPAD) consensus guidelines recommendations 2014^35^. BMI categories had a cut-off at 30, the established limit for obesity^36^. Participants were also dichotomized based on physical work capacity, with a cut-off at 92.5% (i.e. the median of the study population) of predicted maximum workload at exercise test^34^.

### Statistical analysis

Power analysis from the results of a pilot study showed that a sample size of N = 31 per group would detect a difference in left atrial reservoir strain with α = 0.05 and a power of 0.9. Recruiting 44 participants per group would also enable subgroup analysis of 20 individuals although with lesser power (0.7–0.75). Statistical analyses were performed using software system Statistica, version 13. TIBCO Software Inc. (2018). http://tibco.com. Continuous parameters were expressed as means with standard deviations (SD) and medians with range. Mann–Whitney U Test was used to describe the difference in median in all continuous values between groups. Significance level was set at p values < 0.05. p-values in Tables 1, 2, 3 and 4 are shown for descriptive purposes, not inferential. Analysis of variance (ANOVA and ANCOVA) was used, with analysis of disjunct groups such as gender, age categories and groups, with correction for age and/or left atrial volume. Duncan’s Multiple Range test was used. Significance levels of ANCOVA were set at p values ≤ 0.05 and were used for inferential purposes. The strain variables in the study were normally distributed.

**Table 1:** Baseline characteristics of the study population.

Variables	T1DM	Control	p	Total
N	43	43		86
Sex n (%)				
Female	24 (56)	22 (51)		46 (53)
Male	19 (44)	21 (49)	0.669	40 (47)
Age (year)				
Mean (SD)	20 (6)	20 (6)		20 (6)
Median (range)	19 (11–30)	20 (10–30)	0.843	20 (10–30)
Age category median n (%)				
< 18	16 (37)	18 (42)		34 (40)
≥ 18	27 (63)	25 (58)	0.663	52 (60)
Height (cm)				
Mean (SD)	171 (11)	172 (11)		171 (11)
Median (range)	170 (148–192)	173 (144–188)	0.339	172 (144–192)
Weight (kg)				
Mean (SD)	72 (18)	67 (15)		69 (16)
Median (range)	72 (39–134)	66 (32–97)	0.199	68 (32–134)
Body surface area (m^2^)				
Mean (SD)	1.83 (0.25)	1.78 (0.24)		1.80 (0.24)
Median (range)	1.83 (1.32–2.51)	1.79 (1.15–2.14)	0.542	1.82 (1.15–2.51)
Body mass index (kg/m^2^)*				
Mean (SD)	25 (5)	23 (3)		24 (4)
Median (range)	25 (17–41)	23 (19–32)	0.017	24 (17–41)
SBP (mmHg)				
Mean (SD)	116 (12)	117 (10)		116 (11)
Median (range)	115 (95–150)	120 (100–136)	0.275	120 (95–150)
DBP (mmHg)				
Mean (SD)	69 (10)	69 (7)		69 (8)
Median (range)	70 (50–92)	70 (58–84)	0.549	70 (50–92)
Heart rate (beats/min)				
Mean (SD)	73 (12)	67 (11)		70 (12)
Median (range)	70 (52–93)	67 (44–85)	0.039	70 (44–93)
Exercise test (max Watt)				
Mean (SD)	191 (53)	208 (69)		200 (62)
Median (range)	170 (130–333)	184 (100–380)	0.190	180 (100–380)
Exercise test (%)*				
Mean (SD)	92 (14)	97 (17)		94 (16)
Median (range)	93 (57–128)	96 (68–136)	0.302	93 (57–136)

Differences analysed using Mann–Whitney U-test for continuous variables and Fishers exact test for frequencies. Results are expressed as mean with standard deviation (SD) and median with range.

*BSA* body surface area (m^2^), BMI body mass index (kg/m^2^) (corrected for age according to WHO), *SBP* systolic blood pressure, *DBP* diastolic blood pressure, *HR* heart rate (beats/min).

Significance level < 0.05. Exercise test (*Percentage of predicted maximum workload for age, sex and BSA).

**Table 2:** Baseline characteristics for participants with diabetes.

Variables	< 18 years	≥ 18 years	p	Total
N	16	27		0
Age at diabetes onset				
Mean (SD)	6 (3)	11 (4)		9 (4)
Median (range)	6 (1–11)	10 (2–19)	< 0.001	9 (1–19)
Duration of diabetes (years)				
Mean (SD)	9 (3)	13 (5)		11 (5)
Median (range)	8 (4–16)	13 (4–25)	0.008	11 (4–25)
HbA1c (mmol/mol)*				
Mean (SD)	54 (5)	56 (8)		55. (7)
Median (range)	54 (45–65)	57 (43–71)	0.451	55 (43–71)
HbA1c (mmol/mol)**				
Mean (SD)	55 (5)	58 (7)		57 (6)
Median (range)	55 (49–67)	57 (45–74)	0.148	56 (45–74)
Average B glucos 7 days				
Mean (SD)	9.4 (1.2)	9.5 (1.9)		9.4 (1.6)
Median (range)	9.4 (7.2–11.7)	9.1 (7.0–15.3)	0.439	9.2 (7.0–15.3)
Body mass index (kg/m^2^)***				
Mean (SD)	24.7 (5.4)	25.4 (4.6)		25.2 (4.9)
Median (range)	24.3 (17.3–38.4)	24.7 (18.7–40.5)	0.664	24.7 (17.3–40.5)

Differences analysed using Mann–Whitney U-test for continuous variables and Fishers exact test för frequencies. Results are expressed as mean with standard deviation (SD) and median with range.

*HbA1c* glycated haemoglobin (*closest to the time of examination, **over time from onset). Body mass index (kg/m^2^) (***corrected for age according to WHO).

Significance level < 0.05.

**Table 3:** Echocardiographic parameters of left ventricle.

Variables	T1DM	Control	p	Total
N	43	43		86
IVSd (mm)				
Mean (SD)	8 (1)	8 (1)		8 (1)
Median (range)	8 (5–11)	8 (6–10)	0.380	8 (5–11)
LVPWd (mm)				
Mean (SD)	8 (1)	7 (1)		8 (1)
Median (range)	7 (5–11)	7 (5–10)	0.394	7 (5–11)
LVED vol indexed (ml/m^2^)				
Mean (SD)	44 (9)	51 (8)		48 (10)
Median (range)	44 (24–66)	52 (39–70)	< 0.001	47 (24–70)
LV ejection fraction (%)				
Mean (SD)	65 (4)	64 (4)		64 (4)
Median (range)	65 (58–73)	64 (53–72)	0.582	65 (53–73)
E velocity (cm/s)				
Mean (SD)	93 (19)	92 (15)		93 (17)
Median (range)	92 (66–139)	94 (66–123)	0.810	92 (66–139)
A velocity (cm/s)				
Mean (SD)	50 (14)	46 (12)		48 (13)
Median (range)	46 (31–95)	41 (26–77)	0.096	44 (26–95)
E/A (ratio)				
Mean (SD)	1.97 (0.59)	2.14 (0.54)		2.06 (0.57)
Median (range)	1.8 (1.1–3.3)	2.1 (1.3–3.3)	0.101	1.9 (1.1–3.3)
Average e′ vel (cm/s)				
Mean (SD)	14 (2)	15 (2)		15 (2)
Median (range)	14 (9–19)	15 (10–20)	0.108	15 (9–20)
E/e′ (cm/s)				
Mean (SD)	6.8 (1.8)	6.2 (1.0)		6.5 (1.5)
Median (range)	6.7 (4.2–11.8)	6.3 (4.1–9.0)	0.286	6.4 (4.1–11.8)
S/D (ratio)				
Mean (SD)	0.89 (0.26)	0.79 (0.21)		0.84 (0.24)
Median (range)	0.9 (0.4–1.4)	0.8 (0.5–1.6)	0.018	0.8 (0.4–1.6)

Differences analysed using Mann–Whitneys U-test. Results are expressed as mean with standard deviation (SD) and median with range.

*IVSd* intra ventricular septum diastole, *LVPWd* left ventricular posterior wall diastole, *LVED vol* left ventricle end diastolic volume, *LV* left ventricle, *E* early mitral inflow velocity, *A* mitral inflow velocity at atrial contraction, *DecT* deceleration time, *e' vel* mitral annulus early diastolic velocity, *S* systole, *D* diastole, pulmonary venous flow.

Significance level < 0.05.

**Table 4:** Echocardiographic results of left atrial volume and strain.

Variables	T1DM	Control	p	Total
N	43	43		86
Reservoir (peak) strain (%)				
Mean (SD)	39.9 (5.4)	42.8 (4.8)		41.4 (5.2)
Median (range)	39.0 (32.0–55.7)	43.3 (32.3–52.7)	0.005	42.5 (32.0–55.7)
Conduit strain (%)				
Mean (SD)	27.5 (4.8)	30.9 (4.7)		29.2 (5.0)
Median (range)	27.0 (19.3–37.7)	31.7 (20.0–41.0)	0.001	29.2 (19.3–41.0)
Contractile strain (%)				
Mean (SD)	12.4 (2.8)	11.8 (2.6)		12.1 (2.7)
Median (range)	11.3 (7.3–18.0)	12.0 (1.7–17.7)	0.513	12.0 (1.7–18.0)
Maximum LA volume indexed (ml/m^2^)				
Mean (SD)	23.8 (6.2)	27.6 (5.3)		25.7 (6.0)
Median (range)	22.6 (13.4–46.9)	27.4 (18.2–42.3)	< 0.001	25.6 (13.4–46.9)
LASt (%)				
Mean (SD)	0.17 (0.05)	0.15 (0.03)		0.16 (0.04)
Median (range)	0.17 (0.10–0.32)	0.16 (0.08–0.19)	0.023	0.16 (0.08–0.32)

Differences described using Mann–Whitney U-test. Results are expressed as mean with standard deviation (SD) and median with range.

*LA* left atrium, *LASt* left atrium stiffness.

Significance level < 0.05.

### Reproducibility

Intraobserver and interobserver agreement were measured in 25 subjects for LA reservoir strain, calculated by using Dahlberg's analysis, Intraclass Correlation Coefficient (ICC) and the Bland–Altman method. Intravariability with Dahlberg's analysis provided a COV (Smethod/mean) of 2%. The degree of interrater agreement (ICC) showed an estimated variability of k-score mean of 0.989 in a 95% confidence interval from 0.975 to 0.995. Inter variability with Dahlberg's analysis provided a COV (Smethod/mean) of 3.1%. The degree of inter-rater agreement (ICC) showed an estimated variability of k-score mean of 0.976 in a 95% confidence interval from 0.945 to 0.989.

## Results

### Baseline clinical characteristics

A total of 88 individuals participated in the study, 45 with T1DM and 43 healthy controls. Two participants with T1DM were excluded due to insufficient echocardiographic image quality, impeding the analysis of atrial strain (feasibility 97.8%).

Participants with T1DM had significantly higher BMI and higher heart rate than the controls. The higher heart rates were mainly seen in those with a diabetes duration of more than 10 years. Otherwise, no differences were seen in baseline characteristics between the T1DM group and the control group (Table 1). Metabolic characteristics for the T1DM group are presented in Table 2.

### Left ventricular measurements

All echocardiographic results of the LV were within normal range^33,37,38^ and LVEF did not differ between the groups (Table 3). Demonstrated differences in LA-function (see below) remained when corrected for LVED volume (ANCOVA analysis).

Among the traditional diastolic function parameters only the pulmonary venous flow systole (S)/diastole (D) ratio was significantly higher in the T1DM group compared to the control group (descriptive statistics, Table 3). Post hoc ANOVA tests showed that individuals with T1DM ≥ 18 years had significantly higher S/D (although within normal range) than both T1DM and healthy individuals < 18 years, whereas controls ≥ 18 years did not differ significanly in S/D from their younger peers. Other traditional diastolic function parameters (i.e. E-wave velocity, E/A, e' and E/e'), showed no difference between T1DM and control groups.

### Left atrial measurements

The T1DM group had significantly smaller left atrial volumes (both in absolute values and as indexed) compared to the control group (p < 0.05). Indexed left atrial volume correlated negatively with heart rate in the T1DM group (r = − 0.45, p < 0.01). The difference in atrial size between groups remained significant after correction for age, heart rate, isoBMI and physical capacity (percent of predicted maximum workload at exercise test) one at a time, but not when corrected for all covariates at once (p = 0.12). Echocardiographic results of the left atrium are presented in Table 4.

#### Left atrial phasic functions (reservoir, conduit and contractile strain).

**Figure 2:**
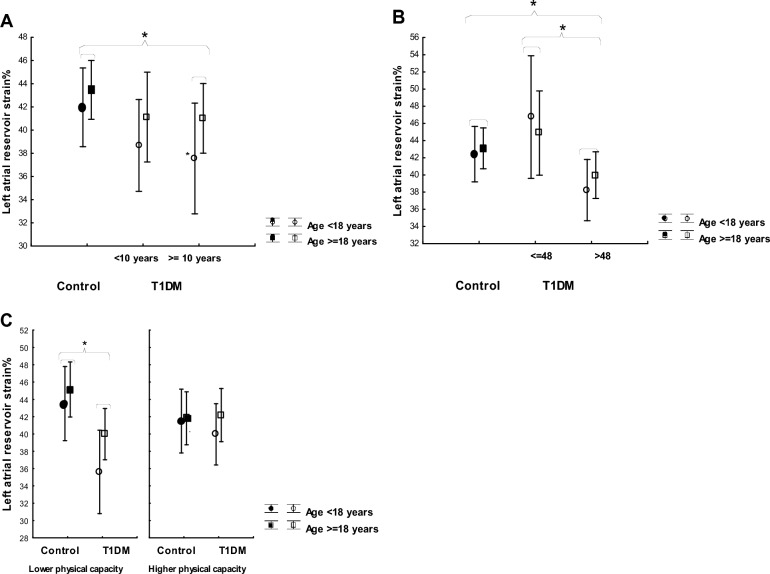
Reservoir strain (%) in control and T1DM subgroups. Results are presented in relation to (**A**) diabetes duration (< 10 and ≥ 10 years), (**B**) HbA1c (≤ 48 and > 48 mmol/mol) and (**C**) physical capacity (≤ 92.5% and > 92.5% (i.e. the median of the population) of predicted maximum workload at exercise test). Two age categories are shown: children (< 18 years) and adults (≥ 18 years). Analyses are corrected for LA volume, and 95% confidence intervals shown.

The individuals with T1DM had significantly lower reservoir and conduit strain than the healthy controls (p < 0.05). Reservoir and conduit strain were found to be significantly lower in the individuals with a diabetes duration of 10 years or longer than in the control group (p < 0.05) and the trend was similar in both genders and in both age categories, i.e. < 18 years and ≥ 18 years of age (Fig. 2a). No significant difference was seen in contractile strain between T1DM and controls. No correlation between left atrial volume and left atrial reservoir strain, nor between heart rate and left atrial reservoir strain, was seen in the T1DM group.

#### Modulating factors of left atrial function

Mean HbA1c levels over time, from onset of disease to examination date, correlated negatively with reservoir (r = − 0.3, p < 0.05) and conduit strain (r = − 0.4, p < 0.05). Individuals with the most recent HbA1c of 48 mmol/mol or below (n = 6) showed a significantly higher reservoir strain (p < 0.01) than their peers with the most recent HbA1c above 48 mmol/mol (n = 37) (Fig. 2b). Average glucose on CGM during the week preceeding the examination correlated negatively with reservoir and conduit strain (r = − 0.4, p < 0.05), and the same pattern was seen in both age categories, i.e. < 18 years and ≥ 18 years of age.

Obesity (BMI ≥ 30) was seen in 16% of T1DM individuals, but only in 2% of controls. T1DM participants with BMI ≥ 30 (n = 7) had significantly lower reservoir strain (p < 0.05) compared to those with BMI < 30 (n = 36). A correlation between BMI and reservoir strain was demonstrated for the entire study population (r = − 0.25, p < 0.05).

The small groups of T1DM individuals with HbA1c ≤ 48 mmol/mol and BMI ≥ 30 were mutually exclusive, but otherwise individuals in both age categories, of both genders, with both high and low physical capacities, and both high and low atrial volumes were represented in both sets of individuals. When these two subgroups were excluded from analyses, the difference between T1DM and control groups in reservoir and conduit strain remained.

There was a significant difference in reservoir and conduit strain between control participants and participants with diabetes with a lower physical capacity (p < 0.05), but not between those with a higher physical capacity (Fig. 2c). Controls with a higher physical capacity displayed slightly lower atrial reservoir strain than their peers with lower physical capacity, while the T1DM group showed the opposite relationship (ANCOVA between effects, p < 0.05).

No reliable association between systolic or diastolic blood pressure and atrial phase function was found.

#### Left atrial stiffness

**Figure 3:**
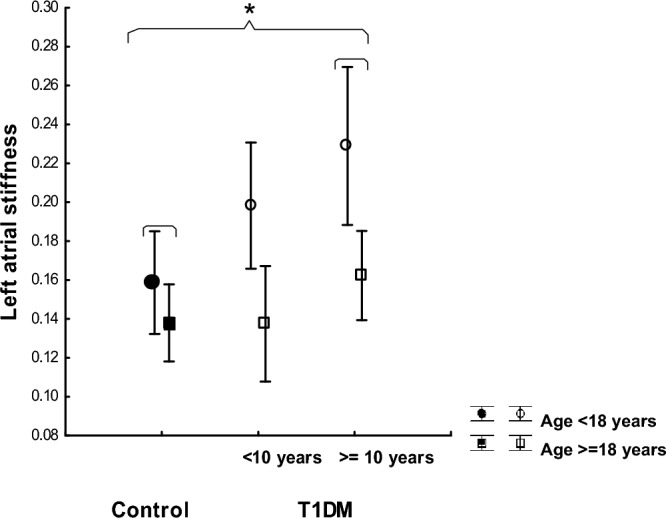
LA-stiffness in controls and in T1DM subgroups for duration of diabetes (< 10 and ≥ 10 years). Two age categories are shown: children (< 18 years) and adults (≥ 18 years). Analyses are corrected for LA volume, and 95% confidence intervals shown.

LA-stiffness proved to be significantly higher in individuals with longer diabetes duration (p < 0.05) than the control group, and individuals < 18 years with T1DM had significantly higher LA-stiffness than individuals ≥ 18 years (Fig. 3). LA-stiffness was significantely higher in the small group of individuals with BMI ≥ 30 (n = 7) (p < 0.05), and there was a positive correlation between IsoBMI and LA-stiffness (r = 0.31, p < 0.05) for the whole population.

## Discussion

Participants with T1DM present with significantly lower atrial reservoir and conduit strain, and significantly higher atrial stiffness than the control group. These results confirm one previous study^5^, but the observation of similar trends in children and adults alike is unique to this study.

Furthermore, only the subgroup of T1DM individuals with lower physical capacity display a lower left atrial function compared to low-performing controls, while there is no difference between high-performing T1DM individuals and controls. This indicates a beneficial effect of physical activity in T1DM in terms of left atrial function, which to our knowledge has not been shown before in individuals as young as the participants of this study.

The participants with T1DM had higher BMI and higher heart rates, as well as smaller left ventricles and atria, compared to the control group. The higher BMI in the T1DM group compared to controls is an expected finding since exogenous insulin affects body composition and/-or eating habits increasing fat accumulation^36^. Higher heart rate is also a common finding in study populations including subjects with T1DM, especially in those with longer diabetes duration^39,40^, likely as part of a disturbed balance between sympathetic and parasympathetic autonomic nerve activity, i.e. cardiac autonomic dysfunction.

The finding that the T1DM group had significantly smaller ventricles and atria than controls is more puzzling. However, smaller volumes could in part be explained by higher heart rates among the subjects with diabetes. An alternative explanation is that controls achieved slightly higher percentages of expected maximum workload at exercise test than T1DM subjects (though ns), implying a possible higher level of physical activity: since physical activity is known to cause remodelling of both ventricles and atria resulting in enlargement^41,42^, this slight skewness in performance between groups might in part explain why the control group had larger volumes than the T1DM group. Finally, a third hypothesis is that the inflammatory process per se in diabetes affects the myocardium, primarily causing remodelling with smaller heart volumes and secondarily leading to higher heart rates^15^. The sizes of the chambers, i.e. the LA and the LV in T1DM vary in the literature^43^; moreover atrial size depend on age and degree of diastolic function where it is well known that more advanced diastolic dysfunction will on the contrary result in a dilatation of the left atrium^44^. In short, there is no definite explanation to smaller LV and LA volumes in T1DM to be found in the literature, apart from the various hypothesis mentioned above. It is possible that this finding might be characteristic of the young study population with T1DM at hand, and represent a transient remodelled state before normalization and, later, dilatation of chambers. Only prospective studies of young populations with T1DM with long term follow-up can answer this question.

When analysing differences in atrial phase function between groups (ANCOVA), we chose to correct for age and left atrial volume as continuous predictors, in order to prevent factors related to body size from affecting the results. We did not correct for heart rate or BMI since these factors are part of the pathophysiology in diabetes. Focusing on atrial function, we chose not to explore LV anatomical features or LV function further in the scope of this article, ascertaining by ANCOVA analysis that LV size did not influence the main outcome of atrial strain, however.

In terms of metabolic control, the sample of participants with T1DM in this study corresponded to the national population of T1DM patients as reported to the Swedish National Diabetes Register for similar age groups, and during the corresponding time period (2019–2021), with only slightly lower HbA1c values than national means (54.1 vs 55 mmol/mol in adolescents, and 58.5 vs 60 mmol/mol in adults^45^).

Reduced left atrial reservoir strain is generally considered a sign of left ventricular diastolic dysfunction^27^, and left atrial phase function is therefore suggested as a useful parameter in the algorithms for assessing heart failure with preserved ejection fraction (HFpEF)^26,28^.

Recent studies have shown that decreased LA strain has both diagnostic and prognostic value for HFpEF^26^ and that LA stiffness is the most reliable index to identify early diastolic dysfunction before the progression to HFpEF^37^. This suggests that LA strain imaging and LA-stiffness index could constitute early and sensitive markers of diastolic dysfunction in T1DM.

Lower LA reservoir strain and higher LA stiffness as demonstrated in this study on a group level, may therefore be interpreted as a possible and subtle early sign of diastolic dysfunction of the left heart, although in a young and asymptomatic population. It was therefore surprising to find significantly higher LA-stiffness in T1DM children and adolescents < 18 years compared to adults ≥ 18 years with T1DM. Since LA-stiffness is calculated as the ratio between E/e’ and reservoir strain this result must be interpreted with caution when involving a paediatric population: Reservoir strain seems to be a robust analysis, while E/e′ is age sensitive and to a certain extent dependent on heart rate, which might serve to amplify the differences in LA-stiffness both between age groups, and between subjects with T1DM and controls. However, the possibility remains that the higher LA-stiffness in the younger age group might in part be due to the significantly earlier onset of disease in this group. On the other hand, in this study individuals with T1DM ≥ 18 years differed from their younger peers in S/D pulmonary flow by pulsed wave Doppler, while controls ≥ 18 years did not. We suggest that individuals with T1DM antecede healthy controls in converting to adult patterns of S/D pulmonary venous flow which implies that T1DM impacts the filling of the left atrium over time.

Currently and to our knowledge only one other study has been conducted to investigate atrial function using LA-strain phasic functions and LA-stiffness in adolescents and young adults with T1DM^5^. Ifuku et al. divided their study population into three age categories and interpreted their results as a sign of progression of LA strain reduction over age groups, with lower reservoir strain seen in adolescents and young adults with T1DM, but not in children with T1DM (< 15 years old), while higher LA stiffness was only seen in adults > 30 years old with T1DM. While our main result confirms that of Ifuku, the younger age group in the present study showed the exact same pattern as the older age group, with lower reservoir and conduit strain and higher LA stiffness. In addition to the earlier onset of disease in those < 18 years, this may be explained by the fact that the younger participants in the present study exhibited a longer duration of diabetes than the children in Ifuku’s study (mean duration of 2.7 (Ifuku) versus 7.2 years for comparable age groups, i.e. < 15 years). The younger group in this study is admittedly more comparable to Ifuku's second age group (15–29 years), which displayed a diabetes duration of 8.3 years. We therefore deduce that, not surprisingly, it is the duration of disease and not age as such that determines the presence of LA function reduction in individuals with T1DM, and that children, too, are subject to the early influences of T1DM on LA function.

Modulating factors with a possible protective effect on left atrial phase function that emerge from this study are lower HbA1c levels, a BMI below 30 and a higher physical capacity.

The HbA1c parameter, which correlated negatively with left atrial function in this study, was based on data from diabetes onset until study examination date, which should be a fair representation of metabolic control over time. Possibly indicating an element of reversibility with improved metabolic control, a most recent HbA1c below 48 mmol/mol seemed beneficient in terms of left atrial function, but the result was based on a few individuals only (n = 6). Howewer, the result was further reinforced by data from CGM with a negative correlation between mean glucose levels the previous week and reservoir strain. The results support the notion that because hyperglycaemia causes an inflammatory processes and endothelial dysfunction in T1DM^15^, hyperglycaemia is likely to be involved in the pathophysiological mechanism of left atrial dysfunction.

Obesity, i.e. BMI ≥ 30, is more prevalent among individuals with T1DM than in the general population^36,46^, and is associated with an increased risk of cardiovascular disease^47^. Left atrial stiffness has been found to be elevated in obese children and in adults (BMI ≥ 30) and to be related to insulin resistance^48^. Even if based on a small number of patients (n = 7), our results are in line with these previous findings, supporting the notion that the double burden of T1DM and obesity is more deleterious in terms of atrial function than T1DM alone, supposedly through mechanisms such as increased peripheral insulin resistance (double diabetes^49^), and altered lipid profiles resulting in lipotoxicity of the heart^50^.

The beneficial effects of physical activity in terms of cardiovascular health have been amply demonstrated, also in young populations and in children^51^. In this study, significant reduction in left atrial strain in individuals with T1DM compared to controls was only seen in the section of the population with a physical capacity below median (92.5%), while T1DM individuals with a physical capacity above median did not differ in left atrial phase performance from controls. It is worth noting that controls and T1DM displayed significantly different relationships between physical capacity and LA strain, where controls with higher physical capacity showed slightly lower left atrial strain than controls with lower physical capacity. In previous publications, left atrial strain at rest in young healthy athletes has been shown to be similar^41^, or decreased^52^, compared to non-athletic healthy controls, which agrees with our observation. Assuming individuals with higher physical capacity exercise to a greater extent than their peers with lower physical capacity, these results would appear to support a notion that physical activity is beneficial in terms of left atrial function for individuals with T1DM, children and young adults alike.

## Limitations

The first and main limitations of this study relate to the small study population size. Whereas the main objective of the study, i.e. demonstrating a significant difference in LA function between subjects with T1DM and healthy controls, was achieved with statistical robustness, the complexity of the material proved such that in order to analyse subgroups and combinations of modulating factors, a larger population size would have been preferable. As a consequence, significant differences in LA-function were demonstrated for the whole study population, and concurrent trends for subgroups of age, diabetes duration and physical capacity were shown. Regarding the very small subgroups of HbA1c and BMI cathegories, results were only included since they were supported by significant correlations in the larger population. Nevertheless these results should be interpreted with caution. A second limitation corncerns the reference range for LA function parameters which only extend down to age 20 in the NORRE study^25^ and down to age 18 in the World Alliance Societies of Echocardiography Study^29^. Normal values cannot be extrapolated to children and adolescents, impeding the assessment of pathology in this group. Finally, selection bias cannot be excluded among those who voluntarily participated in the study: individuals who are physically active are more likely to agree to undergo examinations that include fitness tests, which may be the reason why the control participants in this study had slightly better physical capacity.

## Conclusion

Adolescents and young adults aged 10–30 years with type 1 diabetes had significantly lower reservoir and conduit strain and higher left atrial stiffness than healthy controls. Longer duration of diabetes and lower physical capacity seem to have an adverse effect on left atrial phase function and left atrial stiffness, regardless of the age of the participants. Meanwhile, LA function in individuals with T1DM probably benefits from lower all time HbA1c and may benefit from BMI < 30. Measurements of LA phase reservoir and conduit function and LA-stiffness may become new important tools in assessing heart function and improve the understanding of early diastolic dysfunction in individuals with T1DM, already at an early age. However, further longitudinal follow-up studies are required to confirm these findings.

## Data Availability

The data underlying this article will be shared on reasonable request to the corresponding or the senior author.

## Abbreviations

A: Mitral inflow velocity at atrial contraction
a': Mitral annulus velocity at atrial contraction
BMI: Body mass index
BP: Blood pressure
CI: Confidence interval
D: Diastole
DBP: Diastolic blood pressure
DCM: Diabetic cardiomyopathy
E: Early mitral inflow velocity
e': Mitral annulus early diastolic velocity
EDV: End-diastolic volume
ESV: End-systolic volume
GFR: Glomerular filtration rate
GLS: Global longitudinal strain
HbA1c: Glycated haemoglobin
HFpEF: Heart failure with preserved ejection fraction
ISPAD: International Society for Pediatric and Adolescent Diabetes
IVRT: Isovolumic relaxation time
IVSd: Intra ventricular septum diastole
LA: Left atrium
LAVI: Left atrium volume indexed
LV: Left ventricle
LVED vol: Left ventricle end diastolic volume
LVEF: Left ventricle ejection fraction
LVIDd: Left ventricle internal diameter diastole
LVOT: Left ventricle outlet tract
LVPWd: Left ventricular posterior wall diastole
OR: Odds ratio
PV_a_: Pulmonary venous reverse flow velocity at atrial contraction
PW: Pulsed wave
RVIDd: Right ventricle internal diameter diastole
TDI: Tissue doppler imaging
T1DM: Type 1 diabetes mellitus
s': Mitral annulus systolic velocity
SBP: Systolic blood pressure
S: Systole
STE: Speckle-tracking echocardiography
S_CD: Conduit strain
S_CT: Contractile strain
S_R: Reservoir strain
vel: Velocity
vol: Volume
y: Year
2-ch: Two-chamber
3-ch: Three-chamber
4-ch: Four-chamber
2D: Two-dimensional
3D: Three-dimensional

## References

[1] Rosengren A, Svensson AM, Kosiborod M, Clements M, Rawshani A (2015). Long-term excess risk of heart failure in people with type 1 diabetes: A prospective case-control study. Lancet Diabetes Endocrinol..

[2] Cieluch A, Zozulinska-Ziolkiewicz D (2020). Can we prevent mitochondrial dysfunction and diabetic cardiomyopathy in type 1 diabetes mellitus?. Int. J. Mol. Sci..

[3] Alonso, N., & Mauricio, D. Pathogenesis, clinical features and treatment of diabetic cardiomyopathy. *Adv. Exp. Med. Biol.* **1067**, 197–217. 10.1007/5584_2017_105 (2018). 10.1007/5584_2017_105 28980272

[4] Patterson, C. C. *et al.* Worldwide estimates of incidence, prevalence and mortality of type 1 diabetes in children and adolescents: Results from the International Diabetes Federation Diabetes Atlas, 9th edition. *Diabetes Res. Clin. Pract.* **157**, 107842. 10.1016/j.diabres.2019.107842 (2019). 10.1016/j.diabres.2019.107842 31518658

[5] Ifuku M (2021). Left atrial dysfunction and stiffness in pediatric and adult patients with Type 1 diabetes mellitus assessed with speckle tracking echocardiography. Pediatr. Diabetes.

[6] Álvarez-Almazán S, Filisola-Villaseñor JG, Alemán-González-Duhart D, Tamay-Cach F, Mendieta-Wejebe JE (2020). Current molecular aspects in the development and treatment of diabetes. J. Physiol. Biochem..

[7] Intensive Diabetes Treatment and Cardiovascular Outcomes in Type 1 Diabetes: The DCCT/EDIC study 30-year follow-up. *Diabetes Care* **39**, 686–693. 10.2337/dc15-1990 (2016). 10.2337/dc15-1990 PMC4839174 26861924

[8] Huerta-Uribe N, Ramírez-Vélez R, Izquierdo M, García-Hermoso A (2023). Association between physical activity, sedentary behavior and physical fitness and glycated hemoglobin in youth with type 1 diabetes: A systematic review and meta-analysis. Sports Med..

[9] Vestberg D, Olsson M, Gudbjörnsdottir S, Svensson AM, Lind M (2013). Relationship between overweight and obesity with hospitalization for heart failure in 20,985 patients with type 1 diabetes: A population-based study from the Swedish National Diabetes Registry. Diabetes Care.

[10] Vilarrasa N, San Jose P, Rubio MÁ, Lecube A (2021). Obesity in patients with type 1 diabetes: links, risks and management challenges. Diabetes Metab. Syndr. Obes. Targets Ther..

[11] Corbin KD, Pratley RE, Smith SR, Maahs DM, Mayer-Davis EJ (2018). Obesity in type 1 diabetes: Pathophysiology, clinical impact, and mechanisms. Endocr. Rev..

[12] Vazeou A (2022). Increased prevalence of cardiovascular risk factors in children and adolescents with type 1 diabetes and hypertension: The SWEET international database. Diabetes Obes. Metab..

[13] Tikkanen-Dolenc H (2017). Frequent and intensive physical activity reduces risk of cardiovascular events in type 1 diabetes. Diabetologia.

[14] Wake AD (2022). Protective effects of physical activity against health risks associated with type 1 diabetes: “Health benefits outweigh the risks”. World J. Diab..

[15] Gómez-Perez AM, Damas-Fuentes M, Cornejo-Pareja I, Tinahones FJ (2021). Heart failure in type 1 diabetes: A complication of concern? A narrative review. J. Clin. Med..

[16] Mandavia CH, Aroor AR, Demarco VG, Sowers JR (2013). Molecular and metabolic mechanisms of cardiac dysfunction in diabetes. Life Sci..

[17] Miki T, Yuda S, Kouzu H, Miura T (2013). Diabetic cardiomyopathy: Pathophysiology and clinical features. Heart Failure Rev..

[18] Nemes A, Lengyel C, Domsik P, Kalapos A, Várkonyi TT (2016). Complex evaluation of left atrial dysfunction in patients with type 1 diabetes mellitus by three-dimensional speckle tracking echocardiography: results from the MAGYAR-Path Study. Anatol. J. Cardiol..

[19] Rakha S, Aboelenin HM (2019). Left ventricular functions in pediatric patients with ten years or more type 1 diabetes mellitus: Conventional echocardiography, tissue Doppler, and two-dimensional speckle tracking study. Pediatr. Diabetes.

[20] Ward M (2014). Mechanisms underlying the impaired contractility of diabetic cardiomyopathy. World J. Cardiol..

[21] Salvador DB (2022). Diabetes and myocardial fibrosis. JACC Cardiovasc. Imaging.

[22] Brunvand L, Fugelseth D, Stensaeth KH, Dahl-Jørgensen K, Margeirsdottir HD (2016). Early reduced myocardial diastolic function in children and adolescents with type 1 diabetes mellitus a population-based study. BMC Cardiovasc. Disord..

[23] Wojcik M, Starzyk J (2010). Left ventricular diastolic dysfunction in adolescents with type 1 diabetes reflects the long- but not short-term metabolic control. J. Pediatr. Endocrinol. Metab..

[24] Nielsen AB, Hauser R, Johansen ND, Lassen MCH, Jensen GB (2021). Normal values and reference ranges for left atrial strain by speckle-tracking echocardiography: The Copenhagen City Heart Study. Eur. Heart J. Cardiovasc. Imaging.

[25] Sugimoto T (2018). Echocardiographic reference ranges for normal left atrial function parameters: Results from the EACVI NORRE study. Eur. Heart J. Cardiovasc. Imaging.

[26] Khan MS (2020). Left atrial function in heart failure with preserved ejection fraction: A systematic review and meta-analysis. Eur. J. Heart Fail..

[27] Hoit B (2018). Assessment of left atrial function by echocardiography: Novel insights. Curr. Cardiol. Rep..

[28] Boe ESO (2022). Left atrial strain imaging: ready for clinical implementation in heart failure with preserved ejection fraction. Eur. Heart J. Cardiovasc. Imaging.

[29] Singh A, Miyoshi T, Prado AD, Addetia K, Bellino M (2022). Normal values of left atrial size and function and the impact of age: Results of the world alliance societies of echocardiography study. J. Am. Soc. Echocardiogr..

[30] Morris DA, Aravind-Kumar R, Kropf M, Frydas A, Braunauer K (2018). Potential usefulness and clinical relevance of adding left atrial strain to left atrial volume index in the detection of left ventricular diastolic dysfunction. JACC Cardiovasc. Imaging.

[31] Tadic M, Cuspidi C (2021). Left atrial function in diabetes: Does it help?. Acta Diabetol..

[32] Cole TJ, Lobstein T (2012). Extended international (IOTF) body mass index cut-offs for thinness, overweight and obesity. Pediatr. Obesity.

[33] Lang RM (2015). Recommendations for cardiac chamber quantification by echocardiography in adults: An update from the american society of echocardiography and the European association of cardiovascular imaging. J. Am. Soc. Echocardiogr..

[34] Brudin LJ, Pahlm O (2014). Comparison of two commonly used reference materials for exercise bicycle tests with a Swedish clinical database of patients with normal outcome. Clin. Physiol. Funct. Imaging.

[35] Dimeglio LA (2018). ISPAD Clinical Practice Consensus Guidelines 2018: Glycemic control targets and glucose monitoring for children, adolescents, and young adults with diabetes. Pediatr. Diabetes.

[36] Van Der Schueren B (2021). Obesity in people living with type 1 diabetes. Lancet Diab. Endocrinol..

[37] Kou S, Dulgheru R, Voilliot D, De Sousa C, Kacharava G (2014). Echocardiographic reference ranges for normal cardiac chamber size: Results from the NORRE study. Eur Heart J. Cardiovasc. Imaging.

[38] Díaz A, Zócalo Y, Bia D (2020). Percentile curves for left ventricle structural, functional and haemodynamic parameters obtained in healthy children and adolescents from echocardiography-derived data. J. Echocardiogr..

[39] Kristiansen E (2020). Assessing heart rate variability in type 1 diabetes mellitus—Psychosocial stress a possible confounder. Ann. Noninvas. Electrocardiol..

[40] Metwalley KA, Hamed SA, Farghaly HS (2018). Cardiac autonomic function in children with type 1 diabetes. Eur. J. Pediatr..

[41] Rundqvist L, Faresjö M, Carlsson E, Blomstrand P (2017). Regular endurance training in adolescents impacts atrial and ventricular size and function. Eur. Heart J. Cardiovasc. Imaging..

[42] Letnes JM (2020). Left atrial volume, cardiorespiratory fitness, and diastolic function in healthy individuals: The HUNT study, Norway. J. Am. Heart Assoc..

[43] Zairi IMK, Kamoun S, Moussa FB, Rezgallah R, Maatoug J (2019). Impairment of left and right ventricular longitudinal strain in asymptomatic children with type 1 diabetes. Indian Heart J..

[44] Cioffi, G., & Stefenelli, C. Influence of age on the relationship between left atrial performance and left ventricular systolic and diastolic function in systemic arterial hypertension. *Exp. Clin. Cardiol.* **11**, 305–310 (2006). PMC2274841 18651023

[45] Swedish National Diabetes Register (NDR), *Swediabkids Annual Report.*10.18158/S1t0tko3K (2020).

[46] Hoit B (2014). Left atrial size and function: Role in prognosis. J. Am. Coll. Cardiol..

[47] Pinhas-Hamiel O (2015). Prevalence of overweight, obesity and metabolic syndrome components in children, adolescents and young adults with type 1 diabetes mellitus. Diab. Metab. Res. Rev..

[48] Marlow AL (2019). Young children, adolescent girls and women with type 1 diabetes are more overweight and obese than reference populations, and this is associated with increased cardiovascular risk factors. Diab. Med..

[49] Mahfouz RA, Gomma A, Goda M, Safwat M (2015). Relation of left atrial stiffness to insulin resistance in obese children: Doppler strain imaging study. Echocardiography.

[50] Cleland SJ, Fisher BM, Colhoun HM, Sattar N, Petrie JR (2013). Insulin resistance in type 1 diabetes: what is ‘double diabetes’ and what are the risks?. Diabetologia.

[51] Nakamura K (2022). Pathophysiology and treatment of diabetic cardiomyopathy and heart failure in patients with diabetes mellitus. Int. J. Mol. Sci..

[52] Henriksson HHP, Tynelius P, Ekstedt M, Berglind D, Labayen I (2020). Cardiorespiratory fitness, muscular strength, and obesity in adolescence and later chronic disability due to cardiovascular disease: a cohort study of 1 million men. Eur. Heart J..

[53] Liu M (2022). Left atrial function in young strength athletes: Four-dimensional automatic quantitation study. Int. J. Cardiovas. Imaging.

